# Radiation-induced toxicity after image-guided and intensity-modulated radiotherapy versus external beam radiotherapy for patients with spinal bone metastases (IRON-1): a study protocol for a randomized controlled pilot trial

**DOI:** 10.1186/s13063-017-1847-1

**Published:** 2017-03-03

**Authors:** Eva Meyerhof, Tanja Sprave, Stefan Ezechiel Welte, Nils H. Nicolay, Robert Förster, Tilman Bostel, Thomas Bruckner, Ingmar Schlampp, Jürgen Debus, Harald Rief

**Affiliations:** 1 Department of Radiation Oncology, University Hospital Heidelberg, Im Neuenheimer Feld 400, 69120 Heidelberg, Germany; 2 Department of Medical Biometry, University Hospital Heidelberg, Im Neuenheimer Feld 305, 69120 Heidelberg, Germany; 3 National Center for Radiation Oncology (NCRO), Heidelberg Institute for Radiation Oncology (HIRO), Im Neuenheimer Feld 400, 69120 Heidelberg, Germany

**Keywords:** Spinal bone metastases, Intensity-modulated radiotherapy, Palliative radiotherapy, Toxicity

## Abstract

**Background:**

Radiation therapy (RT) of bone metastases provides an important treatment approach in palliative care treatment concepts. As a consequence of treatment, the extent of radiation-induced toxicity is a crucial feature with consequences to a patient’s quality of life. In this context this study aims at reducing the extent of radiation-induced side effects and toxicity by assuming a better sparing of normal tissue with the use of intensity-modulated instead of conventionally delivered external beam radiotherapy.

**Methods/design:**

In this prospective, randomized, single-center trial for patients with spinal bone metastases, RT is performed as either image-guided intensity-modulated radiotherapy (10x3Gy) or conventionally fractionated external beam radiotherapy (10x3Gy). Afterwards radiation-induced toxicity will be assessed and compared 3 and 6 months after the end of radiation.

**Discussion:**

The aim of this pilot study is the evaluation of achievable benefits, with reduced radiation toxicity being the primary endpoint in the comparison of intensity-modulated radiotherapy versus conventional radiotherapy for patients with spinal bone metastases. Secondarily, bone re-calcification, quality of life, pain relief, spinal instability, and local control will be measured and compared between the two treatment groups.

**Trial registration:**

ClinicalTrials.gov, NCT02832830. Registered on 12 July 2016.

**Electronic supplementary material:**

The online version of this article (doi:10.1186/s13063-017-1847-1) contains supplementary material, which is available to authorized users.

## Background

Secondary bone metastases in patients with cancer are most often located in the vertebral spine [1–4], leading to complications such as bone fractures, neurological impairment, and especially pain [5–10]. Radiation therapy of bone metastases as a palliative oncological treatment concept is supposed to prevent and treat symptoms such as fatigue and pain. As a consequence of treatment, the extent of radiation-induced toxicity should be kept as low as possible, as it may also immensely reduce the patients’ quality of life.

Conventionally fractionated external beam radiotherapy is one of the most important radiotherapeutic treatment options for spinal bone metastases, delivering common doses of 30 Gy and resulting in a significant decrease in pain [11]. Nevertheless, the use of this technique is limited due to unsatisfactory sparing of surrounding normal tissue as well as application of high doses to organs at risk such as the spinal cord. As the spinal cord is centrally localized within the vertebral body and thus in the target volume, even modern techniques will never be able to completely spare this at-risk organ. However, every reduction of the dose delivered to nearby tissue still can lead to a decrease of side effects and thus a higher quality of life. The use of intensity-modulated radiotherapy aims to better spare the surrounding tissue, leading to a reduction of possible radiation-induced side effects [12]. Image,guidance additionally matches the patient’s positioning to the treatment field and thus improves the accuracy of dose distribution [13].

Considering the extent of radiation-induced acute and late side effects as well as toxicity with its contribution to reduced quality of life, this randomized study compares the quantitative amount of toxicity by using the two above-mentioned radiotherapeutic treatment concepts.

## Methods/design

### Recruitment and study design

**Fig. 1:**
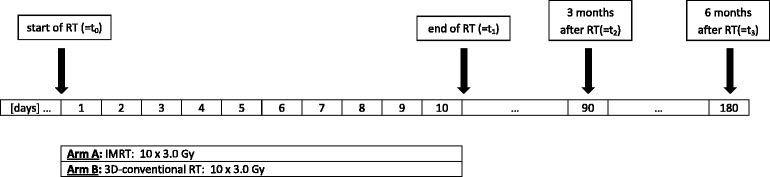
Timeline for IRON-1. *RT* radiotherapy, *IMRT* intensity-modulated radiotherapy

This prospective, single-center pilot study is performed at the Department of Radiation Oncology at the University Hospital of Heidelberg for patients with spinal bone metastases with indication for radiotherapy. As shown in Fig. 1, participants will be randomized into two groups: one group receiving intensity-modulated radiotherapy treatment (group A) and the other conventional external beam radiotherapy (group B).

The Standard Protocol Items: Recommendations for Interventional Trials (SPIRIT) 2013 checklist shows the study guidelines in more detail (see Additional file 1).

Before the start of radiotherapy, the recruitment of eligible participants firstly includes the explanation, to each potential study participant, of the purpose, implementation, design, aims, requirements, and timeline of this study as well as the description of the two different radiation techniques and common ethical issues.

### Inclusion criteria

Patients will be included who:

- Have metastases in the vertebral spine, regardless of number of metastases
- Have an indication for radiation therapy, such as bone pain, spinal instability, and neurological deficit
- Are aged 18 to 85
- Have Karnofsky performance status ≥50
- Have signed a Declaration of Informed Consent

### Exclusion criteria

Patients will be excluded who:

- Have bony lesions caused by multiple myeloma or lymphoma
- Have severe neurological and psychiatric impairment
- Have undergone previous radiation therapy with overlapping areas

### Drop-out criteria

Criteria that will lead to the drop-out of a patient before completion of this study may be any kind of treatment for medical reasons or impairment that will result in an interruption or early completion of radiotherapy. Furthermore, the patient’s desire to exit the study or withdrawal of consent as well as any unexpected serious adverse event (SAE) occurring during treatment or in follow-up that leads to early completion of the study will result in drop-out.

### Radiotherapeutic planning and treatment implementation

All patients receive a planning computed tomography (CT) scan with a slice thickness of 3–5 mm. Patient positioning depends on the area being treated and includes the use of various fixation devices, such as Wingstep® and Prostep® (Elekta, Stockholm, Sweden) for lesions in the thoracic or lumbar spine as well as head holders such as Aquaplast masks (Aquaplast Corporation, Wyckoff, NJ, USA) for cervical spine immobilization. In addition to skin tattoo marks, this guarantees the achievement of a high reproducibility of patient positioning and accuracy of treatment delivery.

Afterwards these CT scans are used to outline organs at risk (OARs) and to contour the clinical target volume (CTV), which encompasses the metastatic vertebral body and, with a margin of 1 cm, results in the planning target volume (PTV). The target volume (=PTV) in each treatment group consists of the entire metastatic vertebral body in a craniocaudal direction including the Proc. costales et transversi to the right and left lateral sides.

In group A, treatment is being performed as intensity-modulated radiotherapy (IMRT) and planned as either tomotherapy, volumetric arc therapy (VMAT), or step-and-shoot IMRT with daily cone-beam CT imaging for rigid patient deformation during radiation. In group B radiation is delivered by conventional, three-dimensional (3D) planned, external beam radiotherapy in two to three fields. Each group uses 6 MV photon energy plans with multileaf collimator shaped radiation fields. A palliative treatment regimen with a total prescribed dose of 30 Gy in 3 Gy fraction doses and daily treatment sessions five times per week is performed, with the 90% isodose surrounding the PTV. OARs such as the skin, heart, or the esophagus are delineated, and dose limit constraints are prescribed to each of them following common considerations according to QUANTEC parameters.

### Data collection and follow-up period

**Fig. 2:**
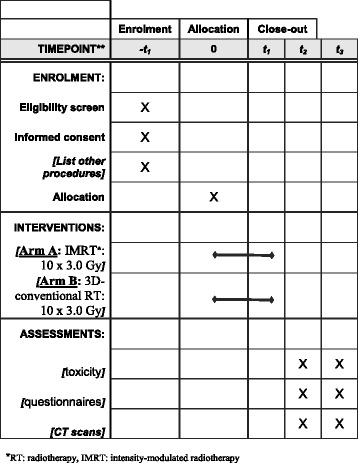
Intervention and assessment schedule for IRON-1 trial

During study observation, patient data are collected before the start of radiotherapy treatment (t0), at the end of treatment (t1), and in the follow-up period at scheduled visits in our department at time points of 3 months (t2) as well as 6 months (t3) after completion of treatment (Figs. 1 and 2).

Patient clinical information is carefully documented in case report forms (CRFs) using questionnaires, filled out by each patient, that measure and quantify clinical symptoms and factors such as pain, radiation-induced side effects and toxicity, as well as quality of life (European Organisation for Research and Treatment of Cancer Quality of Life Questionnaire for patients with Bone Metastases and for measure of Fatigue (EORTC BM22, FA13) and visual analog scale (VAS)). Furthermore, the follow-up requirements include a CT scan 3 and 6 months after completion of radiotherapy for evaluation of treatment response, bone sclerosis, spinal stability, and possible complications such as bone fractures.

### Assessment of the primary and secondary endpoints

In this trial, radiation-induced toxicity 3 months after completion ofpalliative radiotherapy will be evaluated as the primary endpoint. Its extent is measured according to criteria delineated by NCI Common Toxicity Criteria for Adverse Events (CTCAE v 4.0).

Secondary endpoints will be bone re-calcification, quality of life, spinal instability and fractures, local control, and clinical symptoms such as fatigue, pain relief, or neurological deficits.

### Statistical analysis and randomization

This trial is being performed as a pilot study; in the calculation for statistical analysis a total of about 30 patients in each treatment group is considered an appropriate sample size. Due to the explorative character of this study, it is not possible to estimate the total number of cases. However, with a scheduled number of 30 patients per group, it will be possible to detect a standardized mean-value effect (Cohen’s *d*) of 0.8 with a power of 80% and an alpha significance level of 5%. A blocked randomization is used to allocate each participant into group A or B before start of treatment. All variables were analyzed descriptively by tabulation of the measures of the empirical distributions. According to the scale level of the variables, means, standard deviations, and medians as well as minimum and maximum or absolute and relative frequencies, respectively, will be reported. Additionally, for variables with longitudinal measurements, the time courses of individual patients will be analyzed and summarized by treatment groups. Descriptive *p* values of the corresponding statistical tests comparing the treatment groups will be given. The Wilcoxon signed rank test will be used to compare changes in group difference. The Cohen’s effect size (ES) will be assessed for clinically relevant change in questionnaire measures (<0.3 low, 0.3–0.7 moderate, >0.7 strong difference). Moreover patient data are collected in table form at the above-mentioned time points for all completed questionnaires. All statistical analyses will be performed with SAS software v 9.3 or higher (SAS Institute, Cary, NC, USA).

## Discussion

This prospective, single-center pilot trial performed at the Department of Radiation Oncology at the University Hospital of Heidelberg assesses radiation-induced toxicity 3 months after radiotherapy in patients with spinal bone metastases by comparing intensity-modulated radiotherapy versus conventional external beam radiotherapy. Especially in this palliative treatment setting a technical approach to a limited extent of side effects and a higher relief of symptoms is needed to result in higher quality of life for palliative patients. Furthermore, this explorative study analyzes the differences seen in bone sclerosis, quality of life, spinal stability, and local control as well as clinical symptoms such as fatigue, pain relief, or neurological deficits between the two treatment groups.

### Trial status

Patient recruitment has not yet been completed.

## Additional file

Additional file 1: SPIRIT 2013 checklist: recommended items to address in a clinical trial protocol and related documents. (PDF 130 kb)

## Abbreviations

CRF: Case report form
CT: Computed tomography
CTCAE: Common Toxicity Criteria for Adverse Events
CTV: Clinical target volume
GCP: Good Clinical Practice
Gy: Gray
IMRT: Intensity-modulated radiotherapy
OAR: Organ at risk
PTV: Planning target volume
RT: Radiotherapy
SAE: Serious adverse event
VAS: visual analog scale
VMAT: Volumetric arc therapy
